# *Chlorophytum borivilianum* (Safed Musli) root extract prevents impairment in characteristics and elevation of oxidative stress in sperm of streptozotocin-induced adult male diabetic Wistar rats

**DOI:** 10.1186/1472-6882-14-291

**Published:** 2014-08-08

**Authors:** Nelli Giribabu, Kilari Eswar Kumar, Somesula Swapna Rekha, Sekaran Muniandy, Naguib Salleh

**Affiliations:** Department of Physiology, Faculty of Medicine, University of Malaya, 50603 Kuala Lumpur, Malaysia; Pharmacology Division, A.U. College of Pharmaceutical Sciences, Andhra University, Visakhapatnam, 530 003 Andhra Pradesh India; Department of Zoology, Sri Venkateswara University, Tirupati, 517502 Andhra Pradesh India; Department of Molecular Medicine, Faculty of Medicine, University of Malaya, Kuala Lumpur, Malaysia

**Keywords:** *C. borivilianum*, Diabetes mellitus, Sperm, Oxidative stress

## Abstract

**Background:**

We hypothesized that *C. borivilianum* root*,* known to improve male reproductive performance, prevents impairment in characteristics, morphology and elevation of oxidative stress in sperm of diabetics. We therefore investigated the effect of aqueous root extract of *C. borivilianum* on these parameters in diabetic rat model.

**Methods:**

*C. borivilianum* root aqueous extract (250 and 500 mg/kg/day) or glibenclamide (600 μg/kg/day) were administered to streptozotocin (STZ)-induced diabetic male rats for 28 consecutive days. At the end of treatment, animals were sacrificed and sperm were collected. Sperm count and percentages of forward motility, viability, hypoosmotic swelling (HOS) tail-coiled and abnormal sperm were evaluated. Sperm lipid peroxidation product (LPO), hydrogen peroxide (H_2_O_2_) and nitric oxide (NO) levels, total antioxidant capacity (TAC), activity levels of endogenous antioxidant enzymes (superoxide dismutase-SOD, catalase-CAT and glutathione peroxidase-GPx), epididymal sperm density, serum fasting blood glucose (FBG) and HbA1c levels were measured. The expression of sperm caspase-3 was assessed. Meanwhile, i*n-vitro* free radical scavenging activity of *C. borivilianum* root extract was determined and the root extract was analyzed for the presence of bioactive compounds by FTIR spectroscopy.

**Results:**

*C. borivilianum* root aqueous extract prevents the decrease in sperm count, percentages of forward motility, viability, HOS and the increase in abnormal sperm percentage and caspase-3 level in diabetic rats. Sperm LPO, H_2_O_2_ and NO levels, FBG and HbA1c were lower while TAC, SOD, CAT, GPx and epididymal sperm density were higher in diabetic rats receiving *C. borivilianum* root extract treatment. *C. borivilianum* root exhibited strong *in-vitro* free radical scavenging activity which may be due to the phenolic compound.

**Conclusions:**

*C. borivilianum* root extract prevents impairment in sperm characteristics and morphology via preventing elevation of oxidative stress, apoptosis and free radicals levels of the sperm in diabetes. These effects may be achieved through maintaining sperm antioxidant level which could be related to free radical scavenging activity of the root extract by phenolic compounds. These effects could also be due to ability of the extract to maintain near normal serum FBG and HBA1c levels in diabetes.

## Background

Diabetes mellitus (DM), characterized by chronic hyperglycemia resulting from deficiency of insulin secretion, resistance to insulin action or both, is associated with long-term damage, dysfunction and failure of various organs [1]. An alarming high rate of infertility has been reported in diabetic male [2]. Diabetes was found to adversely affect the sperm. Rama Raju et al., [3] reported that diabetic men have high percentage of sperm nuclear DNA fragmentation and apoptosis. Meanwhile, Thakur et al., [4] reported that hyperglycemia in rats diminished sperm count, seminal fluid fructose and antioxidant enzymes. A recent study by Battacharya et al., [5] reported that husbands of infertile couples with diabetes possess lower volume of ejaculates, sperm count and percentage of motile sperm as compared to healthy husbands of couples with proven fertility.

Safed Musli or *Chlorophytum borivilianum* (family: Lilliaceae) is a highly valued medicinal plant in India and is considered as “white gold” in Indian system of Ayurveda [6]. This plant has been reported to possess a number of biological activities including antimicrobial, anti-inflammatory, antipyretic, hepatoprotective, antioxidant, hypolipidemic and antidiabetic [7]. Traditionally, *C. borivilianum* root has been used to treat male impotence, oligozoospermia and erectile dysfunction [8]. Kenjale et al., [9] reported that oral administration of 250 mg/kg/day of *C. borivilianum* root aqueous extract to normal, healthy male rats preserved sperm count while Thakur et al., [10] reported that oral administration of 200 mg/kg/day of this plant extract to adult hyperglycemic male rats provides protection against sexual dysfunction as indicated by high frequency mounting, intromission and ejaculation.

Oxidative stress-induced sperm damage has been suggested as an important pathological mechanism underlying male infertility [11]. The sperm plasma membrane is susceptible to oxidation due to high content of polyunsaturated fatty acids (PUFA) [12]. Lipid peroxidation in sperm membrane can cause various impairments including decreased sperm motility [13]. The sperm could be protected from oxidative damage by endogenous antioxidant enzymes which are found both in sperm [14] and seminal fluid [15]. Meanwhile, the decrease in seminal fluid antioxidant level was reported to contribute to male infertility in humans [16].

We hypothesized that *C. borivilianum* root prevents impairment in sperm characteristics and morphology, oxidative stress and apoptosis in diabetic rats. This study therefore aimed to investigate *C. borivilianum* root extract effect on these sperm parameters in diabetes and to extent the previous observations in normal male rats whereby oral administration of this plant root extract improved male reproductive capability [9, 10].

## Methods

### Drugs and chemicals

Streptozotocin was purchased from Sigma Aldrich (St. Louis, MO, USA). All other chemicals used were of analytical grade.

### Plant collection and preparation of plant extract

Dried roots of *C. borivilianum* were procured from Nandan Agro Farms Pvt. Ltd. (Hyderabad, Andhra Pradesh, India) and authenticated by senior botanist, Madhava Setty, Sri Venkateswara University, Tirupati, India. The plant was deposited at Herbarium with the number: KLU 96568. The dried roots were cut into small pieces and grounded into fine powder. The root powder (1000 g) was subjected to cold maceration in 2 L of sterile distilled water for 48 hours at room temperature, filtered into a clean round bottom flask using adsorbent cotton wool and a filter paper (Whatman No. A-1). The whole process was repeated seven times to ensure maximum yield of water soluble compounds from the root powder. The combined aqueous extract was concentrated at 37°C using a rotary evaporator (R-210, Buchi Labortechnik AG, Flawil, Switzerland) and lyophilized by using a cyodos freeze dryer (Telstar, Barcelona, Spain) to yield approximately 38 g of solid extract (3.8% w/w).

### Phytochemical screening

A qualitative phytochemical evaluation was performed on the aqueous root extracts to determine the presence of carbohydrates (Barfoed’s test), flavonoids (test of Shinoda), phytosterols (Libermann Buchard test), phenols (ferric chloride test), alkaloids (Dragendorff test), proteins (Biuret test) and saponins (Saponification test) following the methods as described by Harbourne [17].

### *Fourier*transform infrared (FTIR) spectroscopy

FTIR spectroscopic analysis was performed using Perkin Elmer spectrophotometer system (PerkinElmer, Inc., Shelton, CT, USA) which detects the characteristic peaks and functional groups that are present in the root extract. The spectral range 4000 to 600 cm^-1^ with resolution of 2 cm^-1^ was used to record the infrared spectra with the potassium bromide (KBr) pellet making technique.

### Experimental animals

Adult male Wistar rats (170–200 g) were obtained from Animal House, Faculty of Medicine, University of Malaya (UM**)**. The rats were kept under standard environmental conditions of room temperature 25 ± 2°C, relative humidity between 45-55% and 12 hrs light/dark cycle. Animals were fed with standard feed pellets (Harlan diet, UK) and tap water *ad libitum*. Experimental procedures were in accordance with ARRIVE guidelines (Animals in Research: Reporting *In-Vivo* Experiments) and European Community Guidelines/ EEC Directive, 1986. This study was approved by the Faculty of Medicine, Animal Care and Use Committee (ACUC), University of Malaya with ethics number: 2013-07-15/FIS/R/NS. Toxicity study was conducted according to Organization for Economic Cooperation and Development (OECD) revised up-and-down procedure for acute toxicity testing (OECD guideline 425) [18]. No signs of toxicity were observed in the tested animals up to a dose of 3000 mg/kg/day.

### Induction of diabetes

Hyperglycemia was induced in overnight fasted male rats by a single intraperitoneal injection of STZ dissolved in ice cold citrate buffer (0.1 M, pH 4.5) at a dose of 55 mg/kg [19]. The rats were allowed to drink 5% sucrose solution overnight after injection, to overcome drug-induced hypoglycemia. Diabetes was confirmed by the presence of polydipsia, polyuria and weight loss and only animals exhibiting a fasting glucose level greater than 300 mg/dL three days after STZ injection were used in this study. Treatment with *C. borivilianum* was commenced four days after STZ injection and this was considered as day one. *C. borivilianum* root extract at 250 and 500 mg/kg/day [20] were administered in the form of suspension in 1% sodium carboxymethylcellulose (Na-CMC) dissolved in distilled water daily for 28 consecutive days using an oral gavage tube.

### Experimental design

The animals were randomly assigned into five experimental groups with six (6) rats per group:

- Group I - Control rats- received 1% Na-CMC vehicle only.
- Group II - Diabetic control rats- received 1% Na-CMC vehicle only.
- Group III and IV - Diabetic rats treated with *C. borivilianum* root aqueous extract at 250 & 500 mg/kg body weight respectively.
- Group V- Diabetic rats treated with standard antidiabetic agent, glibenclamide at 600 μg/kg body weight.

At the end of experimental period, all rats were fasted overnight, weighted and sacrificed under pentobarbital sodium anesthesia (60 mg/kg) followed by cervical dislocation.

### Determination of sperm characteristics and morphology

Immediately after euthanasia, cauda epididymis were dissected out, chopped and placed in 5 ml physiological saline (0.9% NaCl) and incubated for 5 min at 37°C in water bath to allow sperm to leave the epididymal tubules.

#### Evaluation of sperm forward motility

Progressive sperm motility was evaluated by a method of Belsey et al. [21]. Firstly, immotile sperm were counted followed by motile sperm. Sperm forward motility was expressed as percentage of motile sperm to total sperm counted.

#### Sperm count

Sperm count was determined using Neubauer chamber (Deep 1/10 mm, LAMBART, Darmstadt, Germany) of hemocytometer following the method as described by Belsey et al. [21]. Sperm count was expressed as number of sperm per ml of solution.

#### Sperm viability

The ratio of live to dead sperm was determined using 1% trypan blue staining following the method as described by Talbot and Chacon [22]. A total number of 200 sperm were counted per slide and the results were expressed as percentage of the live sperm.

#### Hypo-osmotic Swelling Test (HOST)

Sperm’s flagella membrane integrity was assessed by hypo-osmotic swelling test (HOST). In brief, assay was performed by incubating 50 μL sperm suspension with 1 mL hypo-osmotic solution. Two hundred sperm were evaluated and percentage of live sperm (with coiled tail) was calculated following the method of Jeyendran et al. [23].

#### Sperm morphological abnormalities

Percentages of sperm head, middle piece and tail abnormalities were determined from a total of 300 sperm per rat [24]. Sperm morphology was viewed under a light microscope (Nikon, H600L, Tokyo, Japan) under 400 × magnifications. Data was expressed as percentage of morphologically abnormal sperm to total sperm count.

### Biochemical analyses

Following analysis of sperm parameters, the remaining cauda epididymal sperm suspension was centrifuged at 800 g for 20 min at 4°C and the pellet was resuspended in normal saline. The sperm were homogenized with a glass-Teflon Homogenizer (Heidolph Silent Crusher M, Germany). The supernatant collected was used for biochemical studies.

### Assessment of sperm lipid peroxidation

The lipid peroxidation level in sperm homogenate was measured as malondialdehyde (MDA), which is the end product of lipid peroxidation, which reacts with thiobarbituric acid (TBA) to produce TBA reactive substance (TBARS), a red colored complex which has peak absorbance at 532 nm, according to the method of Ohkawa et al., [25].

### Determination of Hydrogen peroxide and nitric oxide level in sperm

In the supernatant levels of H_2_O_2_ were estimated according to the method by Pick [26]. In brief, H_2_O_2_ mediates horseradish peroxidase-dependent oxidation of phenol red and the results obtained were expressed as nmol H_2_O_2_ formed g^-1^ tissue. Nitric oxide (NO) concentration was measured as nitrite/nitrate by previous method of Miranda et al., [27]. The NO levels were expressed as nmol/g tissue.

### Assessment of Sperm Total Antioxidant Capacity (TAC)

The total antioxidant capacity of sperm homogenate was measured according to the modified method of Erel, [28], where the samples were mixed with acetate buffer, and 2,2’-azino-bis(3-ethylbenzothiazoline-6-sulfonic acid) (ABTS reagent) with hydrogen peroxide. The color reaction was monitored spectrophotometrically at 660 nm. The results were expressed as μmolequivalents Trolox/mg protein.

### Determination of sperm endogenous antioxidant enzyme activity

SOD activity level was assayed according to the method by Misra and Fridovich [29] and was expressed as the amount of enzyme that inhibits oxidation of epinephrine by 50%, which was equal to 1U per milligram of protein. CAT activity level was determined on the basis of H_2_O_2_ decomposition [30] and was expressed in μmol of H_2_O_2_ metabolized/mg protein/min. Meanwhile, GPx activity level was determined according to the method by Rotruck et al. [31] and was expressed as μmol of GSH consumed/mg protein/min.

### *In-vitro*radical scavenging activity of *C. borivilianum*root extract

Antioxidant power of *C. borivilianum* root aqueous extract was evaluated by using DPPH, superoxide, hydroxyl, H_2_O_2,_ NO and reducing power scavenging assays. Ascorbic acid (10–200 μg/ml) was used as a standard. The ability of extract to scavenge or inhibit free radicals was expressed as % inhibition and was calculated using this formula.


A_o_ = absorbance of control group (without plant extract) and A_t_ = absorbance of *C. borivilianum* root aqueous extract. Absorbance was measured using a spectrophotometer (UV-1700, Shimadzu, Kyoto, Japan) at different wavelengths depending on the type of assay performed. All assays were carried out in triplicate.

Radical-scavenging activity of *C. borivilianum* root aqueous extract against stable DPPH radical was determined according to the method by Katalinic et al., [32]. Measurement of superoxide radical scavenging activity was based on the method by Xiang and Ning [33]. Hydroxyl radical scavenging activity was measured using a modified method by Halliwell et al., [34]. The ability of *C. borivilianum* root aqueous extract to scavenge H_2_O_2_ was determined according to the method by Ruch et al., [35]. The ability of the extract to scavenge nitric oxide was determined using a method by Dastmalchi et al., [36]. Finally, reducing power scavenging activity of *C. borivilianum* root extract was determined according to the method by Suseela et al., [37].

### Estimation of sperm caspase-3 levels

Snapped-frozen sperm were homogenized using a sonicator with PRO-PREP (iNtRON Biotechnology, Seoul, South Korea) extraction solution in the presence of protease inhibitors. Total cell protein was obtained by centrifugation at 13000 g for 15 minutes at 4°C. After determination of the protein concentration, the same amount of protein was loaded into the 12% SDS-PAGE gel. The protein was then transferred onto the polyvinyilidene difluoride (PVDF) membrane and incubated in 5% BSA for 90 minutes. The blot was then exposed to primary antibody, caspase-3 rabbit polyclonal IgG, Santa Cruz, USA (sc-7148) at 1:1000 dilution. Following primary antibody incubation, the blot was incubated with HRP-conjugated secondary antibody and finally visualized by using Optic 4C (Bio RAD). β-actin (Santa cruz, sc-130656) was used as a loading control. Photos of the blots were captured and density of each band was determined using Image J software (1.39, Bethesda, Maryland, USA). The ratio of caspase-3/ β-actin band density was determined and was considered the expression level of each of the target proteins.

### Determination of Serum HbA1c and Fasting Blood Glucose (FBG) Levels

At the end of 28 day treatment, blood was withdrawn from retro-orbital plexus in overnight fasted diabetic rats. Glucose levels were estimated using glucose oxidase/peroxidase kit (BioSystems S.A. Costa Brava 30, Barcelona, Spain) and HbA1c was measured using a commercially available kit (BioSystems S.A. Costa Brava 30, Barcelona, Spain). The values were estimated using an automated analyzer Dimension RxL Max Integrated Chemistry System (Siemens Healthcare Diagnostics Inc. Deerfield, IL, USA).

### Epididymal sperm density

Caput epididymis was freshly harvested and fixed in 10% formaldehyde for 24 hours. The tissue was dehydrated in ethanol, cleared in xylene and embedded in paraffin at 58°C. The embedded tissue samples were cut into 5 μm thickness using a microtome (Histo-line laboratories, ARM-3600, Viabrembo, Milano, Italy). After deparaffinization by immersion into xylene for 20 min, tissues were dropped in ethanol solution of decreasing concentrations (100%, 95%, 90% and 80%) for 5 min. Sections were then stained with hematoxylin and eosin (H&E). Sperm density in epididymal lumen was graded as normal (+++), moderately decreased (++), or severely decreased (+) according to description by Narayana et al., [38].

### Statistical analysis

Statistical differences were evaluated by analysis of variance (ANOVA) and Student’s t-test. A probability level of less than 0.05 (p < 0.05) was considered as significant. *Post-hoc* statistical power analysis was performed for all the experiments and values > 0.8 were considered as adequate. Meanwhile, Shapiro-Wilk test was performed to test for data normality and values > 0.05 indicated that data were normally distributed.

## Results

### Phytochemical screening

Qualitative phytochemical screening of aqueous extract of *C. borivilianum* root showed the presence of carbohydrates, proteins, phytosterols and saponins (data not shown).

### FTIR spectral analysis

**Figure 1:**
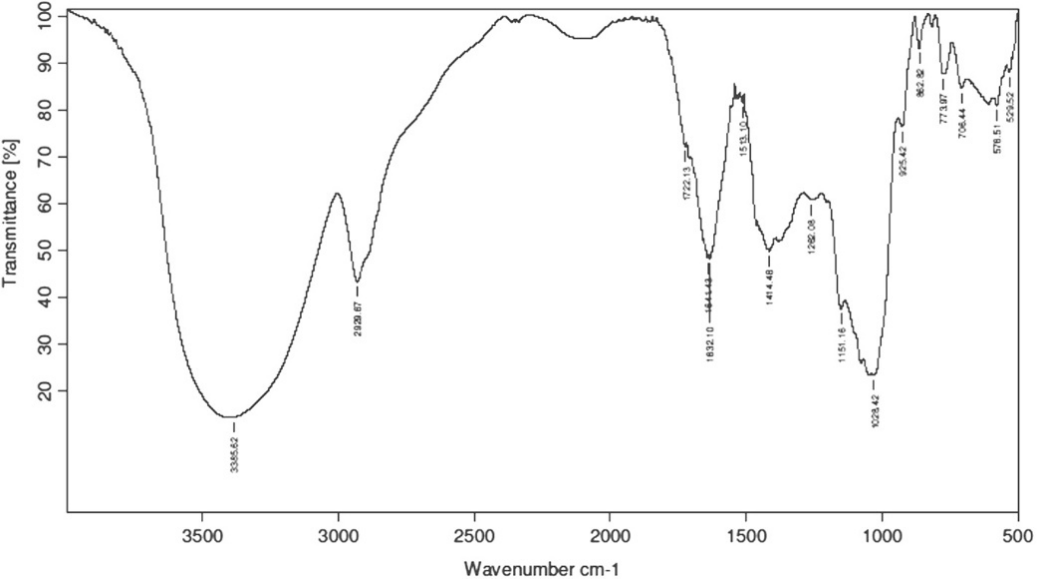
**FTIR spectrum of aqueous extract of** ***C. borivilianum*** **root.** A broad trough is seen at 3385.62 cm^-1^.

Figure 1 shows FTIR spectrum and Table 1 shows the active compounds present in the extract. FTIR spectroscopy is widely used technique in the characterization and identification of different solid-state forms. The FTIR spectra O-H (*Asym str*) of the compound observed at 3385.62 cm^-1^ and also CH_2_ = CH- asymmetric stretching frequency of compound observed at 2929.67 cm^-1^. A weak band was observed at 1720 cm^-1^ indicating that the compound may contain –C = O stretching frequency. Aromatic -C = C (*Str*) peaks are resonating at 1414, 1513, 1632, 1640 cm^-1^. Secondary alcohol C-O stretching frequencies of the compound were observed at 1151 cm^-1^. A strong band of aromatic ether linkage was observed at 1028 cm^-1^ indicating that the compound contained C-O-C functional group and 706–773 cm^-1^ indicating that the compound might be mono substituted.

**Table 1:** **FTIR peak values of extracts of** ***C. borivilianum*** **root extract**

S. No	Area	Functional group	Name of the group
1	3385.62 cm^-1^ (broad trough )	OH (Alcohol) /Phenol	Alcohol/Phenol (High concentration)
2	2929.67 cm^-1^ (medium-strong )	CH_2_ = CH-	Methylene
3	1722.13 cm^-1^ (weak band)	(May be 80%)	Saturated aliphatic/cyclic 6-membered
4	1414,1513,1632,1640 cm^-1^	C = C (Aromatic ring)	Aromatic C = C bond
5	1262 cm^-1^	Aromatic ether linkage (-O-Aromatic ring)	Aromatic ether linkage (phenol derivative)
6	1151 cm^-1^ (weak band)	Alcohol (secondary alcohol)	Alcohol
7	1028 cm^-1^ (strong band)	Aromatic ether stretching frequency	Aromatic ether linkage
8	706-773 cm^-1^	Mono substituted benzene	Mono substituted aromatic ring

### Effect on sperm characteristics

Table 2 shows the effect of treatment of *C. borivilianum* root extract on sperm characteristics in diabetic rats. Our findings indicate that in diabetic rats, sperm count was 41.67% lower than normal, non-diabetic rats. In diabetic rats, total sperm count was approximately 45.87 ± 6.87 million/ml which was lesser than the normal count. 28-days treatment with the root extract of *C. borivilianum* at 250 and 500 mg/kg/day caused a significantly higher count (34.46% and 49.64% respectively) as compared to non-treated diabetic rats. Glibenclamide treatment resulted in 51.55% higher count as compared to non-treated diabetic rats. No significant difference in the count was noted between treatment with 500 mg/kg/day *C. borivilianum* and glibenclamide.

**Table 2:** **Effect of** ***C. borivilianum*** **root extract on sperm parameters**

Parameters	Normal	Diabetic	Diabetic		
			250 mg/kg ***C. borivilianum***	500 mg/kg ***C. borivilianum***	600 μg/kg glibenclamide
Sperm count (millions/ml)	78.64 ± 6.74	45.87^*^ ± 6.87	61.68^†^ ± 5.86	68.64^†^ ± 5.68	69.52^†^ ± 5.71
Sperm motility (%)	73.76 ± 5.84	43.68^*^ ± 6.79	58.75^†^ ± 4.38	68.47^†^ ± 5.76	69.68^†^ ± 5.61
Sperm viability (%)	82.69 ± 4.47	62.87^*^ ± 3.86	71.34^†^ ± 4.79	73.35^†^ ± 4.08	75.38^†^ ± 5.35
HOS tail coiled sperm (%)	69.58 ± 3.64	43.27^*^ ± 3.78	54.86^†^ ± 4.37	61.78^†^ ± 3.61	62.42^†^ ± 4.13

Values are expressed as Mean ± SD of 6 rats, n = 6 per treatment group. ^*^p < 0.01 as compared to control, ^†^p < 0.05 as compared to non-treated diabetic rats.

Meanwhile, sperm motility in diabetic rats was 40.78% lower than normal non-diabetic rats. 28-days treatment with *C. borivilianum* root extract resulted in a significantly higher progressive sperm motility (34.50% and 56.75% respectively) as compared to non-treated diabetic rats. Treatment with 600 μg/kg/day glibenclamide resulted in 59.52% higher progressive sperm motility as compared to non-treated diabetic rats. Glibenclamide was 1.73 times more potent than 250 mg/kg/day *C. borivilianum* root extract in causing an increase in sperm progressive (forward) motility in diabetic rats.

The sperm viability was 23.97% lower in diabetic rats than normal, non-diabetic rats. 28-days treatment with *C. borivilianum* root extract resulted in a significantly higher viability (13.47 and 16.67% respectively) as compared to non-treated diabetic rats. Meanwhile, treatment with glibenclamide resulted in 19.9% higher viability as compared to non-treated diabetic rats. Glibenclamide treatment was 1.48 times more potent than 250 mg/kg/day *C. borivilianum* root extract in causing increased in sperm viability in diabetic rats.

The HOS tail coiled sperm was 37.82% lower in diabetic rats than normal, non-diabetic rats. 28-days treatment with 250 and 500 mg/kg/day *C. borivilianum* root extracts resulted in 26.79% and 42.87% higher HOS tail-coiled sperm respectively as compared to non-treated diabetic rats. Treatment with glibenclamide resulted in 44.26% higher HOS tail-coiled sperm as compared to non-treated diabetic rats. Glibenclamide treatment was 1.65 times more potent than 250 mg/kg/day *C. borivilianum* in causing increased percentage of HOS tail coiled sperm in diabetic rats.

### Effect on sperm morphology and percentages of abnormal sperm

**Figure 2:**
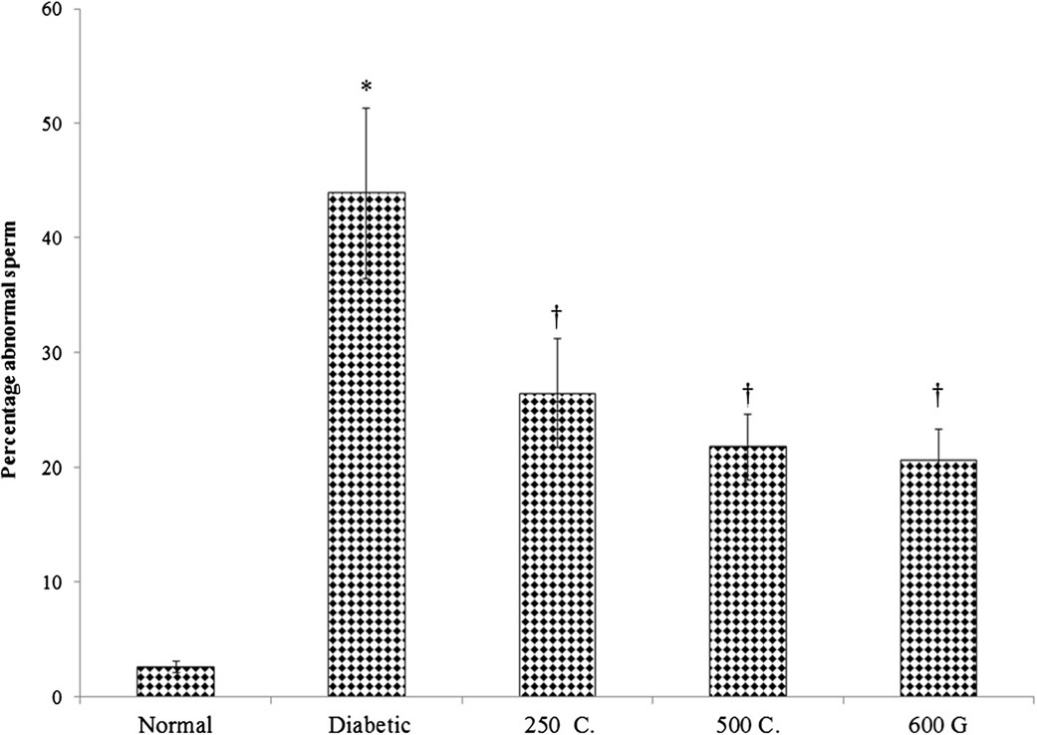
**Percentage of abnormal sperm in different experimental groups.** A significantly higher abnormal sperm percentage was observed in diabetic rats. Treatment with *C. borivilianum* root extract at both doses or glibenclamide lower the percentage of abnormal sperm as compared to non-treated diabetic rats. ^*^p < 0.05 as compared to normal (control), ^†^p < 0.01 as compared to diabetic group. n = 6 rats per group. 250C: 250 mg/kg/day *C. borivilianum* extract, 500C: 500 mg/kg/day *C. borivilianum* extract, 600G: 600 μg/kg/day glibenclamide.

**Figure 3:**
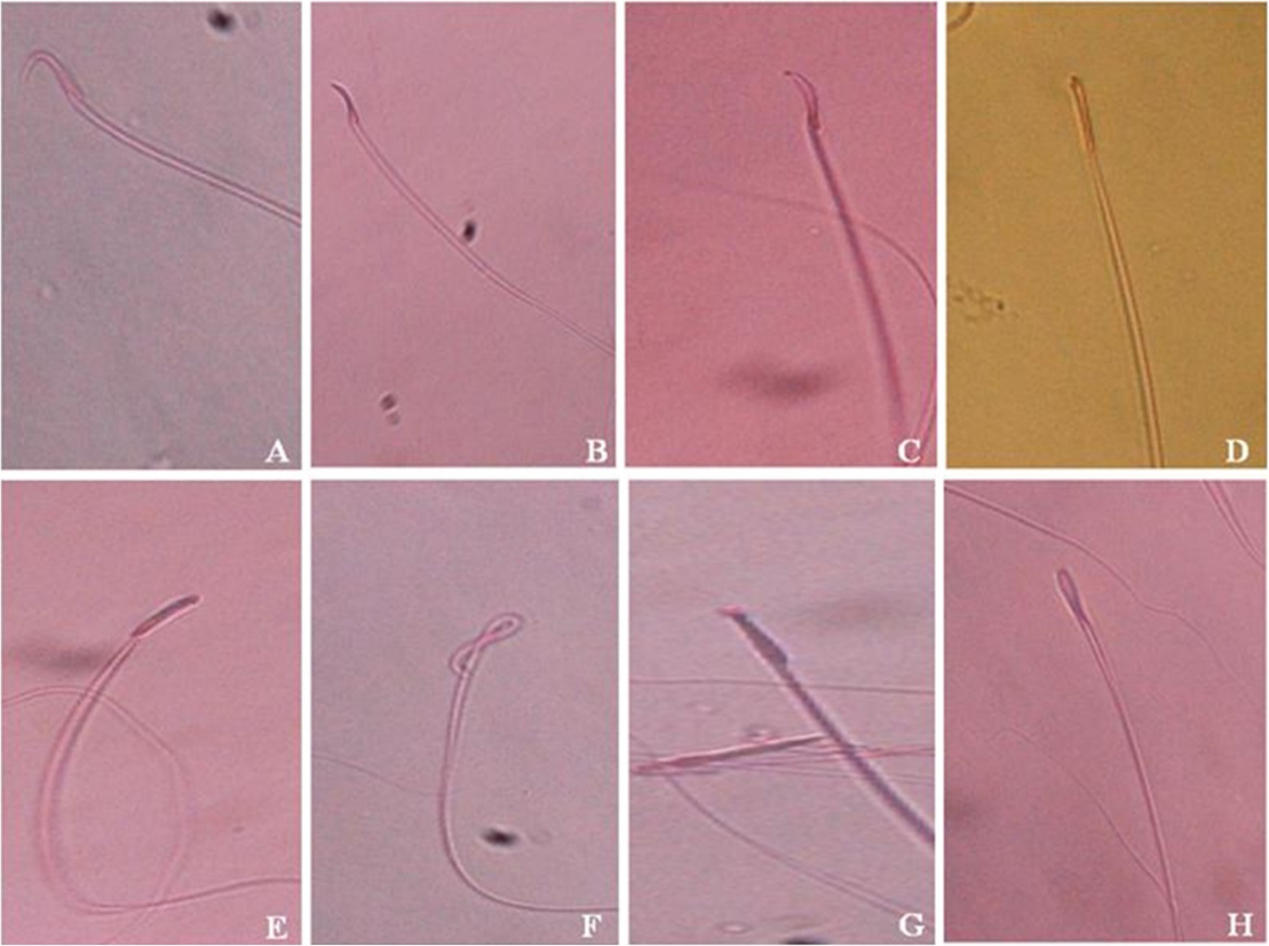
**Appearance of abnormal sperm.** Various abnormal sperm shapes are observed under a light microscope in normal, diabetic rats and diabetic rats treated with *C. borivilianum* or glibenclamide which include **A)** Normal **B)** small hook **C)** thin elongated head **D)** rod head **E)** banana head **F)** bent neck **G)** pin head **H)** macro cephalous; Stained with eosin-nigrosin, 100 × magnification.

Figure 2 shows percentages of abnormal sperm in various treatment groups while Figure 3 shows various sperm morphologies observed in normal, non-diabetic and diabetic male rats. The percentage of abnormal sperm in diabetic rats was 1614.15% higher than in normal, non-diabetic rats. 28-days treatment with 250 and 500 mg/kg/day *C. borivilianum* root extracts resulted in 39.76% and 50.35% lower abnormal sperm percentages respectively as compared to the non-treated diabetic rats. Treatment with glibenclamide resulted in 53.09% lower abnormal sperm percentage as compared to non-treated diabetic rats. Glibenclamide treatment was 1.35 times more potent than 250 mg/kg/day *C. borivilianum* in causing decreased in percentage of abnormal sperm in diabetic rats.

The abnormal sperm morphologies include small hook, thin elongate head, rod head, banana head, bent neck, pin head and macro cephalous (Figure 3). In Table 3, the percentage of each of these abnormal-shaped sperm was shown. Our findings indicate that a significantly higher percentage of pin head and bent neck sperm were observed in diabetic rats, (8.01 and 2.05 times higher respectively) as compared to normal non-diabetic rats. Other sperm shapes such as banana head, rod head, small hook, thin elongated and macro-cephalous were only seen in diabetic rats and not in normal non-diabetic rats. Meanwhile, in diabetic rats, the highest percentage of abnormal shaped sperm is banana head, which accounts for nearly 13.2% followed by rod head, pin head, small hook, bent neck, thin elongated and macro-cephalous. Treatments with *C. borivilianum* root extract at 250 and 500 mg/kg/day to diabetic rats significantly lowered the percentage of abnormal-shaped sperm. Treatment with 500 mg/kg/day *C. borivilianum* caused 87.59%, 82.08%, 77.95%, 75.82% , 58.53%, 44.15% and 42.81% lower in percentages of macrocephalous, thin elongated, rod head, bent neck, small hook, pin head and banana head sperm respectively as compared to non-treated diabetic rats. Glibenclamide treatment caused a significantly lower percentage of rod head and macro cephalic sperm as compared to treatment *with C. borivilianum* root extract however no significant different in percentages were noted between pin head and bent neck sperm in non-treated diabetic rats.

**Table 3:** **Percentage of different types of abnormal sperm in different experimental groups**

Parameters	Normal	Diabetic	Diabetic		
			250 mg/kg ***C. borivilianum***	500 mg/kg ***C. borivilianum***	600 μg/kg glibenclamide
Pin-head sperm	1.03 ± 0.02	8.63^*^ ± 1.05	5.78^†^ ± 0.26	4.82^†^ ± 1.09	8.35^†^ ± 1.06
Banana-head sperm	-	13.15 ± 2.24	8.65 ± 1.09	7.52 ± 1.05	5.89 ± 0.85
Rod less sperm	-	9.84 ± 1.34	3.16 ± 0.22	2.17 ± 0.07	3.53 ± 0.16
Pin less sperm	-	6.39 ± 1.58	2.85 ± 0.11	2.65 ± 0.04	5.18 ± 0.27
Folded tail sperm	1.53 ± 0.03	3.35^*^ ± 0.37	2.59^†^ ± 0.06	0.81^†^ ± 0.06	3.66^†^ ± 0.34
Other shaped sperm	-	1.15 ± 0.18	1.25 ± 0.05	1.34 ± 0.05	2.08 ± 0.05

Values are expressed as Mean ± SD of 6 rats, n = 6 per treatment group. ^*^p < 0.01 as compared to control, ^†^p < 0.05 as compared to non-treated diabetic rats.

### Effect on sperm MDA content

**Figure 4:**
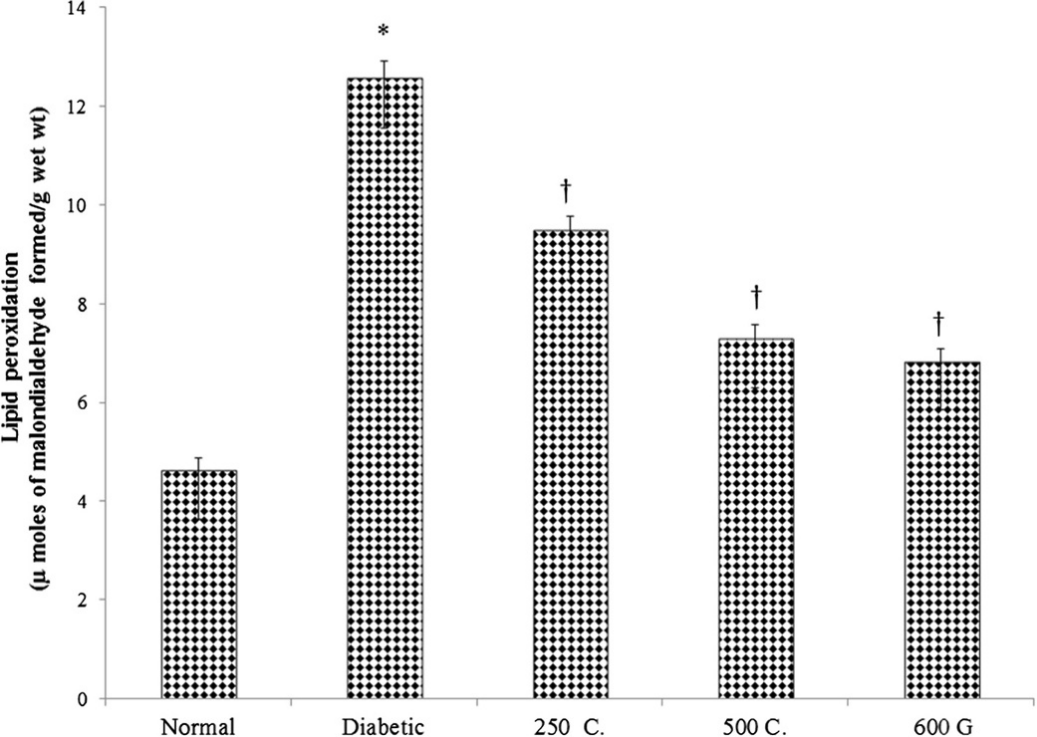
**Sperm MDA content.** Higher MDA level was noted in diabetic rats as compared to normal, non-diabetic rats. Administration of *C. borivilianum* root extract resulted in decreased MDA level. 250C: 250 mg/kg/day *C. borivilianum* root extract; 500C: 500 mg/kg/day *C. borivilianum* root extract, 600G: 600 μg/kg/day glibenclamide. n = 6 per treatment group, ^*^p < 0.05 as compared to normal, non-diabetic control rats, ^†^p < 0.05 as compared to non-treated diabetic rats.

Figure 4 shows the amount of MDA in sperm which was the highest in diabetic rats (171.27%) as compared to normal, non-diabetic rats. Treatment with 250 and 500 mg/kg/day root extract of *C. borivilianum* resulted in 24.44% and 41.96% decrease in MDA content respectively as compared to non-treated diabetic rats. Meanwhile glibenclamide treatment resulted in 45.94% decrease in sperm MDA content as compared to non-treated diabetic rats.

### Effect on hydrogen peroxide and nitric oxide level in sperm

**Figure 5:**
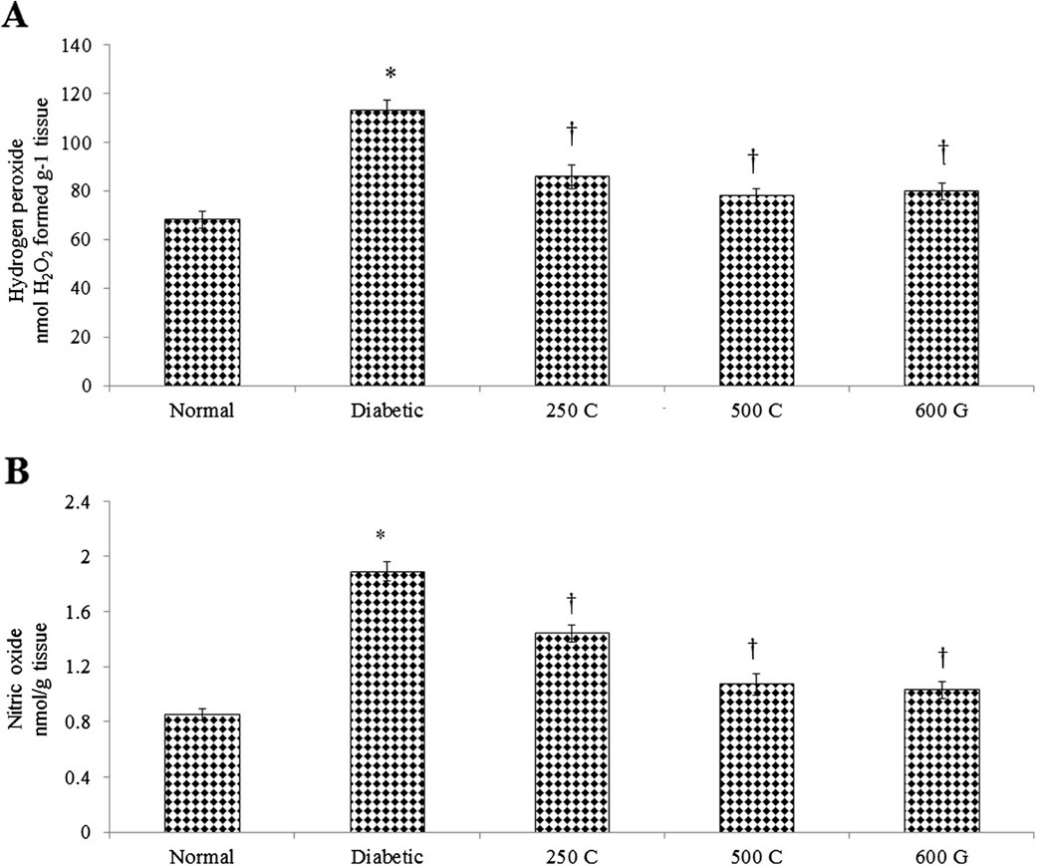
**Sperm H**_**2**_ **O**_**2**_ **and NO levels.** Higher **(A)** H_2_O_2_ and **(B)** NO levels were observed in diabetic rats as compared to normal, non-diabetic rats. Administration of *C. borivilianum* root extract resulted in decreased H_2_O_2_ and NO levels. 250C: 250 mg/kg/day *C. borivilianum* root extract; 500C: 500 mg/kg/day *C. borivilianum* root extract, 600G: 600 μg/kg/day glibenclamide. n = 6 per treatment group, ^*^p < 0.05 as compared to normal, non-diabetic control rats, ^†^p < 0.05 as compared to non-treated diabetic rats.

Figure 5 shows the (A) hydrogen peroxide and (B) nitric oxide levels in sperm which were highest in diabetic rats. Administration of *C. borivilianum* root extract at 250 and 500 mg/kg/day resulted in decreased level of both free radicals. Similar effect was observed following administration of glibenclamide.

### Effect on Sperm Total Antioxidant Capacity (TAC)

**Figure 6:**
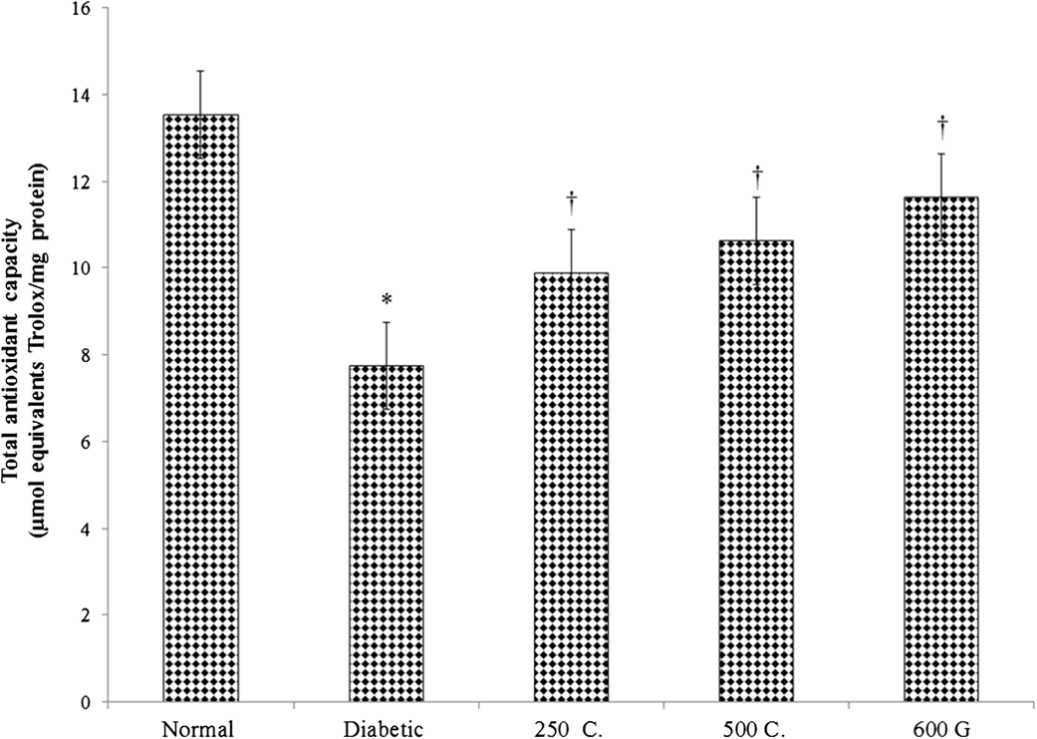
**Sperm total antioxidant capacity (TAC).** TAC was reduced in diabetic rats as compared to normal, non-diabetic rats. Administration of *C. borivilianum* root extract at 250 and 500 mg/kg/day and glibenclamide in diabetic rats resulted in higher TAC as compared to non-treated diabetic rats. 250C: 250 mg/kg/day *C. borivilianum* root extract; 500C: 500 mg/kg/day *C. borivilianum* root extract, 600G: 600 μg/kg/day glibenclamide. n = 6 per treatment group, *p < 0.05 as compared to normal, non-diabetic control rats, †p < 0.05 as compared to non-treated diabetic rats.

Figure 6 shows sperm TAC level which was the highest in normal, non-diabetic rats. The TAC level in diabetic rats was 42.76% lower than normal, non-diabetic rats. Treatment with 250 and 500 mg/kg/day aqueous root extract of *C. borivilianum* resulted in 21.56% and 37.16% higher TAC level as compared to non-treated diabetic rats. Meanwhile, glibenclamide treatment resulted in 50.19% higher TAC level as compared to non-treated diabetic rats.

### Effect of *C. borivilianum*on sperm endogenous antioxidant enzymes

In Table 4, SOD, CAT and GPx activity levels were lowest in diabetic rats. Treatment with 250 and 500 mg/kg/day *C. borivilianum* root aqueous extract resulted in a significantly higher activity levels of SOD, CAT and GPx as compared to non-treated diabetic rats. Similarly, GPx activity level following the root extract treatment was significantly greater than non-treated diabetic rats.

**Table 4:** **Effect of** ***C. borivilianum*** **on SOD, CAT and GPx levels in the sperm of different experimental groups**

Parameters	Normal	Diabetic	Diabetic		
			250 mg/kg ***C. borivilianum***	500 mg/kg ***C. borivilianum***	600 μg/kg glibenclamide
Superoxide dismutase (units/mg protein/min)	1.56 ± 0.06	0.95^*^ ± 0.07	1.26^†^ ± 0.08	1.42^†^ ± 0.06	1.45^†^ ± 0.07
Catalase (H_2_O_2_ metabolized/mg protein/min)	0.64 ± 0.06	0.34^*^ ± 0.08	0.45^†^ ± 0.06	0.52^†^ ± 0.05	0.56^†^ ± 0.07
Glutathione peroxidase (μmol of GSH consumed/ mg protein/min)	1.72 ± 0.05	1.26^*^ ± 0.08	1.57^†^ ± 0.05	1.64^†^ ± 0.06	1.66^†^ ± 0.07

The value represents means ± S.D. for 6 rats per group. ^*^p < 0.01 compared to normal, non-diabetic rats, ^†^p < 0.01 as compared to non-treated diabetic rats.

### *In-vitro*free radical scavenging activity of *C. borivilianum*root extract

**Figure 7:**
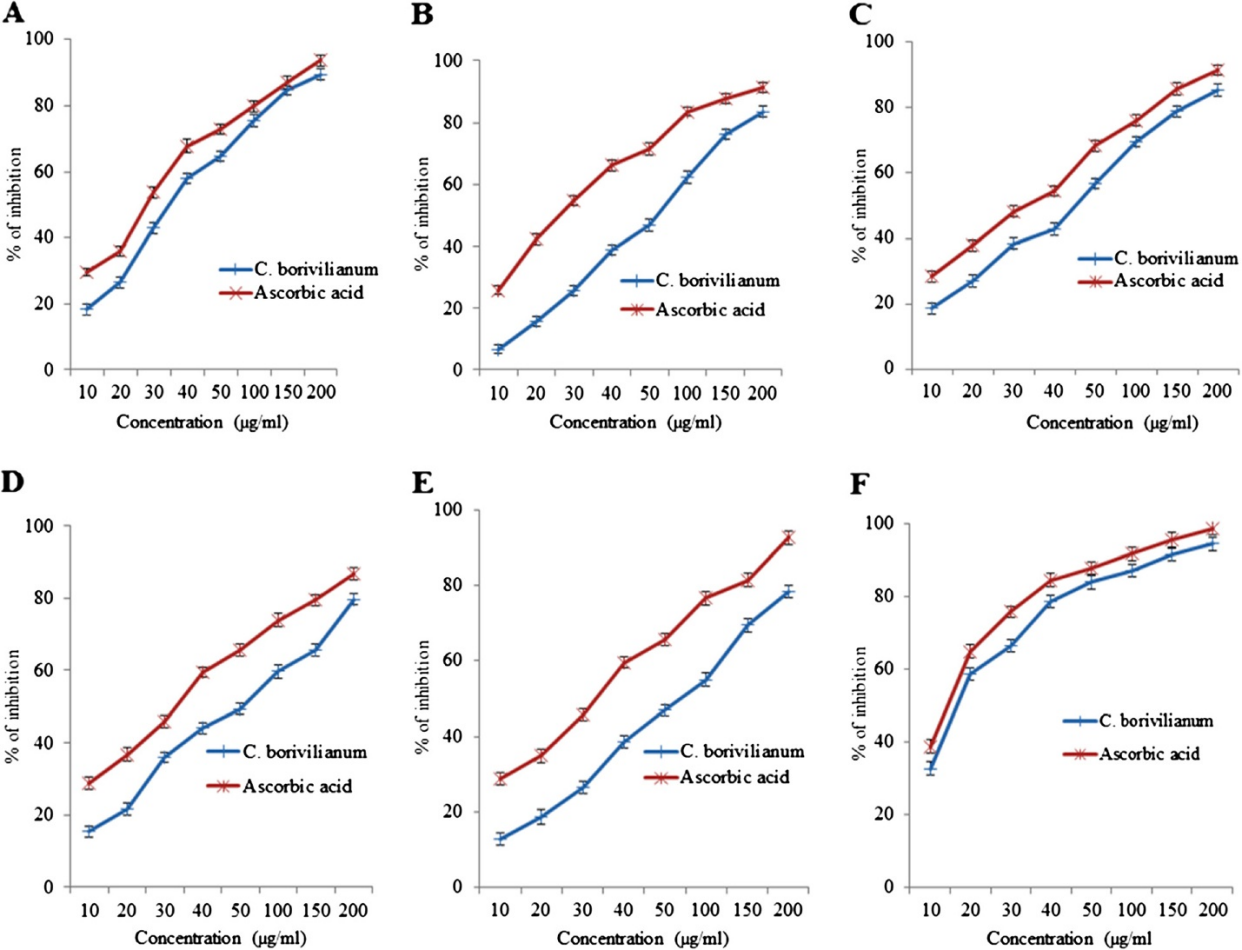
***In-vitro*** **anti-oxidant assay of** ***C. borivilianum*** **root extract.** The graphs showing **A)** DPPH radical; **B)** superoxide radical; **C)** hydroxyl radical; **D)** H_2_O_2_; **E)** nitric oxide and **F)** reducing power scavenging activities of the aqueous root extract of *C. borivilianum* and ascorbic acid. Results represent mean of triplicates for each concentration. For each assay, IC_50_ value for *C. borivilianum* root extract was slightly lower than ascorbic acid.

In Figure 7(A), inhibition of DPPH radicals by 10 μg/ml *C. borivilianum* root extract was 18.36% while for ascorbic acid was 29.65%. IC_50_ for *C. borivilianum* root extract and ascorbic acid were 34.76 μg/ml and 28.47 μg/ml respectively. The IC_50_ for the root extract was 1.22 fold lower than ascorbic acid. Both standard and extract recorded a gradual dose-dependent increase in DPHH radicals inhibition. Figure 7(B) shows the superoxide radical scavenging activity of the root extract of *C. borivilianum* and ascorbic acid which indicate a dose-dependent increase. The percentage inhibition by 10 μg/ml *C. borivilianum* root extract and ascorbic acid were 6.68% and 25.87% respectively. IC_50_ for *C. borivilianum* root extract was 75.45 μg/ml while for standard ascorbic acid was 28.58 μg/ml. The IC_50_ for the root extract was 2.64 fold lower than ascorbic acid. Superoxide anion is a reduced form of molecular oxygen, which plays an important role in the formation of reactive oxygen species (ROS) including H_2_O_2_, hydroxyl radical or singlet oxygen [39].

Figure 7(C) shows dose–response curve for hydroxyl radical scavenging activity of the root extract of *C. borivilianum* and ascorbic acid. IC_50_ for *C. borivilianum* root extract was 44.92 μg/ml while for ascorbic acid was 31.87 μg/ml. The IC_50_ for the root extract was 1.41 times lower than ascorbic acid. Hydroxyl radical is a major active oxygen species formed in the biological system with capability to conjugate with nucleotides in the DNA resulting in strand breakage which could lead to various diseases. Moreover, hydroxyl radical is one of the rapid initiator of lipid peroxidation process via extracting hydrogen atom from the unsaturated fatty acids [40]. Figure 7(D) illustrates a dose-dependent increase in H_2_O_2_ scavenging activity of *C. borivilianum* root extract and ascorbic acid. IC_50_ of the root extract of *C. borivilianum* was 51.82 μg/ml while for ascorbic acid was 32.86 μg/ml. The IC_50_ for ascorbic acid was higher than the root extract by 1.58 times. H_2_O_2_ is a weak oxidizing agent that directly inactivates few enzymes via oxidation of essential thiol (-SH) group. This molecule could rapidly transverses the cell membrane. Once within the cell, H_2_O_2_ can react with Fe^2+^and /or Cu^2+^ to form hydroxyl radical [41].

Our results showing nitric oxide scavenging activity of *C. borivilianum* root extract and ascorbic acid standard are presented in Figure 7(E). The root extract of *C. borivilianum* root exhibits a dose-dependent increase on nitric oxide scavenging activity. IC_50_ for the root extract and ascorbic acid were 65.32 μg/ml and 31.45 μg/ml, respectively. IC_50_ of *C. borivilianum* root extract was lower than ascorbic acid by 2.08 times. NO is a free radical with a single unpaired electron, formed from L-arginine by the action of NO synthase [42]. In the present study, the root extract of *C. borivilianum* showed a significant radical scavenging effect on nitric oxide. Finally, the reducing power scavenging activity of the root extract and ascorbic acid are shown in Figure 7(F). At 10 μg/ml, the percentage inhibition of *C. borivilianum* root extract and ascorbic acid were 32.68% and 38.67% respectively. IC_50_ of the root extract was 18.46 μg/ml while that of ascorbic acid was 13.54 μg/ml. Reducing power IC_50_ of *C. borivilianum* root extract was lower than ascorbic acid by 1.36 times.

### Fasting blood glucose and serum HbA1c levels

Table 5 shows treatment with *C. borivilianum* root extract at 250 and 500 mg/kg/day resulted in 44.5% and 49.2% lower serum FBG and 28.25% and 45.42% lower serum HbA1c levels respectively as compared to non-treated diabetic rats.

**Table 5:** **FBG and serum HbA1c levels in different experimental groups**

Parameters	Normal	Diabetic	Diabetic		
			250 mg/kg ***C. borivilianum***	500 mg/kg ***C. borivilianum***	600 μg/kg glibenclamide
Fasting blood glucose levels (mg/dl)	93.15 ± 6.78	424.16^*^ ± 7.23	235.24^†^ ± 6.78	216.73^†^ ± 4.84	203.68^†^ ± 8.46
Glycosylated hemoglobin, HbA1c (%)	3.46 ± 0.39	9.38^*^ ± 0.72	6.73^†^ ± 0.61	5.17^†^ ± 0.56	5.12^†^ ± 0.43

Values are expressed as Mean ± SD of 6 rats, n = 6 per treatment group. *p < 0.01 as compared to control, †p < 0.05 as compared to non-treated diabetic rats.

### Epididymal sperm density assessment

**Figure 8:**
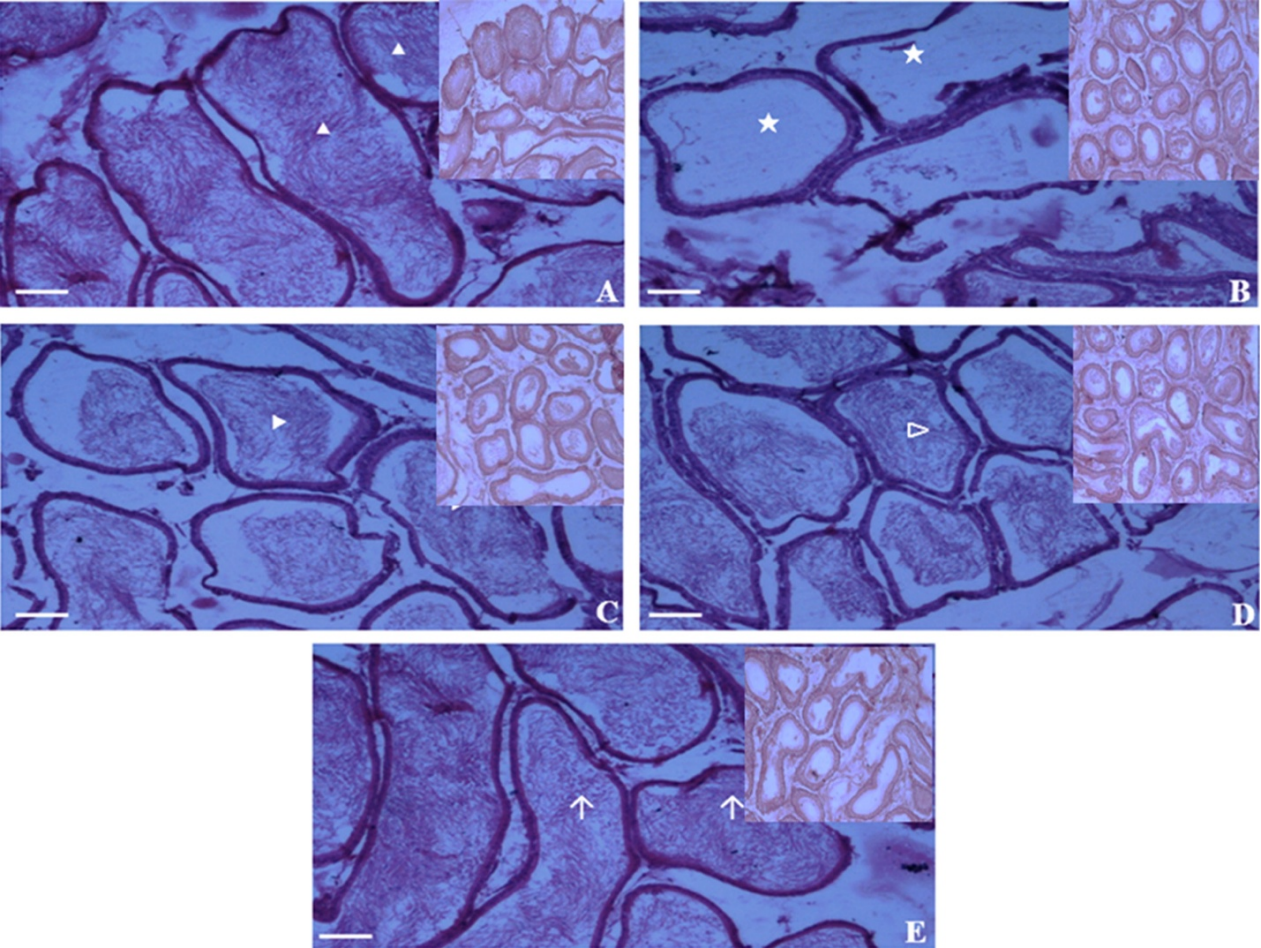
**Morphology of epididymal lumen.** Cross-sections of caput epididymis under high power field (100 ×) and under low power field (20 ×) (upper right hand border) in **(A)** normal, **(B)** STZ-induced diabetic rats **(C)** diabetic rats treated with 250 mg/kg/day *C. borivilianum* extract **(D)** diabetic rats treated with 500 mg/kg/day *C. borivilianum* extract **(D)** and **(E)** glibenclamide. Images showed that most tubules in STZ-induced diabetic rats were devoid of sperm with the tubules appearing smaller in size. Treatment with *C. borivilianum* root extract resulted in higher sperm density and size of the tubular lumens.

Figure 8 shows appearance of epididymal lumen at high and low magnifications in normal non-diabetic, diabetic and diabetic rats treated with different doses of *C. borivilianum* root extract or glibenclamide while Table 6 shows semi-quantitative analysis of sperm density in the epididymal lumens. The highest density was seen in normal, non-diabetic rats. Meanwhile, low sperm density was seen in epididymal lumen of diabetic rats. Some of the lumens were completely devoid of sperm. An increased density was observed following treatment with increasing doses of *C. borivilianum* extract or glibenclamide as compared to non-treated diabetic rats.

**Table 6:** **Assessment of epididymal sperm density**

Parameters	Normal	Diabetic	Diabetic		
			250 mg/kg ***C. borivilianum***	500 mg/kg ***C. borivilianum***	600 μg/kg glibenclamide
Epididymis sperm density	+++	+	++	++	++

+++ Normal density, ++ moderately decreased density, + severely decreased.

### Caspase-3 level in sperm homogenates

**Figure 9:**
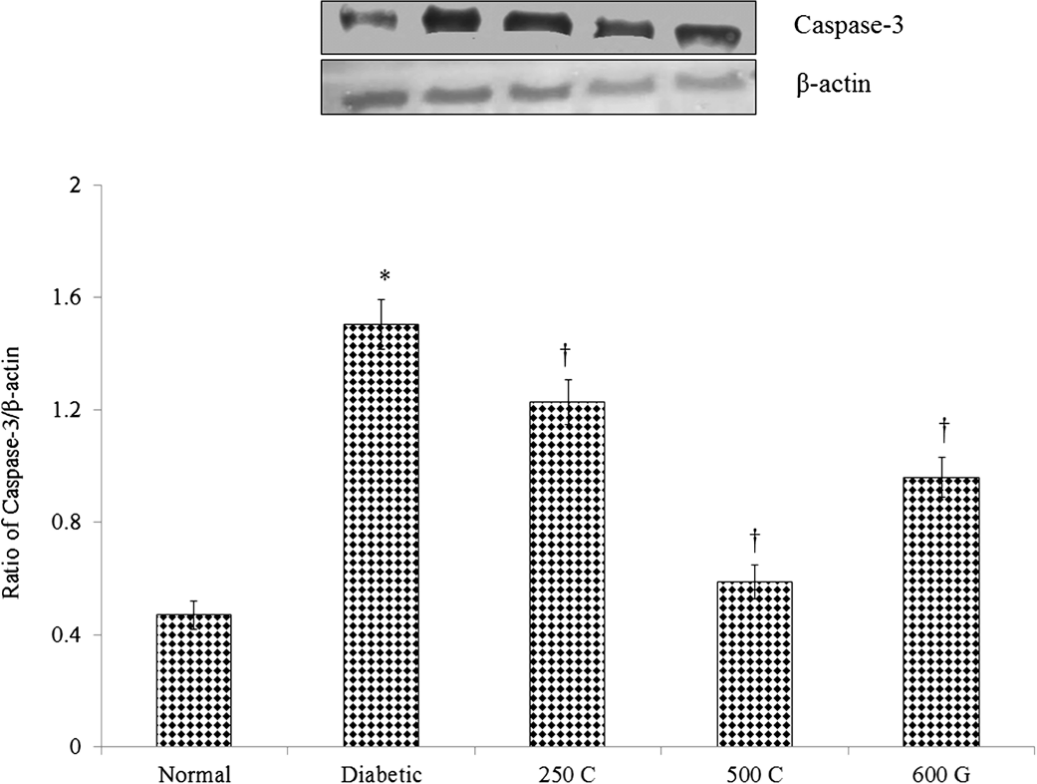
**Expression of Caspase-3 protein in sperm.** Administration of *C. borivilianum* root extract at 250 and 500 mg/kg/day or glibenclamide to diabetic rats resulted in lower expression of caspase-3 protein as compared to non-treated diabetic rats. 250C: 250 mg/kg/day *C. borivilianum* root extract; 500C: 500 mg/kg/day *C. borivilianum* root extract, 600G: 600 μg/kg/day glibenclamide. n = 4 bands per treatment group, *p < 0.05 as compared to normal, non-diabetic control rats, †p < 0.05 as compared to non-treated diabetic rats. MW of caspase-3 is 32 kDa and β-actin is 43 kDa.

Figure 9 shows the effect of *C. borivilianum* aqueous root extract treatment on caspase-3 expression in sperm which was significantly reduced as compared to non-treated diabetic rats. Similarly, glibenclamide administration resulted in a parallel decrease in caspase-3 level of expression.

## Discussion

To the best of our knowledge, this study reported for the first time the effect of root extract of *C. borivilianum* on sperm characteristics and oxidative stress in diabetic rats. Higher sperm count, percentages of sperm forward motility, viability and HOS tail-coiled with lower percentage of abnormal sperm were observed following *C. borivilianum* root extract treatment to STZ-induced diabetic rats. Administration of the root extract also lower the percentage of sperm with abnormal appearances, and in few occasion, the herbal root extract effect exceeds glibenclamide, a reference anti-diabetic drug [43]. Our findings have further shown that *C. borivilianum* root extract was able to lower the level of sperm oxidative stress as evident from lower amount of lipid peroxidation product, MDA, tissue superoxide and nitric oxide free radicals and higher amount of total antioxidant capacity (TAC) and levels of endogenous antioxidant enzymes (SOD, CAT and GPx) in the sperm of diabetic rats. Treatment with *C. borivilianum* root extract also prevents the increase in serum level of FBG and HbA1c in diabetic rats where this might help to prevent negative effects of hyperglycemia on sperm.

An evaluation of sperm characteristics is useful when investigating the underlying cause of male infertility [44]. In the present study, *C. borivilianum* root extract administration to diabetic rats prevented or reduced impairment in sperm characteristics, abnormal sperm percentages and abnormal appearances of sperm. The effect of diabetes on these sperm end point parameters was consistent with other reports in both rats and humans [45–47]. In diabetic rats, average sperm count of approximately 45million/ml was lower than normal (approximately 60 milllion/ml per ejaculate) [19, 48, 49]. Oligozoospermia could predispose diabetic males to subfertility or infertility [50]. The observed decrease in sperm count was supported by diminished sperm intensity in the epididymal lumen. Treatment with *C. borivilianum* root extract has resulted in higher sperm count and epididymal sperm density which suggests that this herb protect the sperm against diabetes-induced damage. In this study, lower percentage of forward moving sperm was also observed in diabetic rats as compared to normal, non-diabetic rats. Sperm with normal forward motility are capable of swimming through the female reproductive tract and ultimately fertilized the oocyte. This characteristic is largely acquired during sperm transit and storage in the epididymis [51]. Several factors including cAMP, intracellular pH and Ca^2+^ level could affect ability of the sperm to display these characteristics [52]. Further studies are required to investigate oxidative stress parameters in testis, epididymis and seminal fluid which would help to ascertain their contribution towards the increase in sperm oxidative stress which subsequently caused sperm damage.

The percentage of sperm viability was also reduced in diabetic rats consistent with reports in both rodents [51, 53] and humans [54]. Kanter et al., [55] reported that in rats, diabetes could induce sperm apoptosis which resulted in reduced sperm viability. Similarly, higher caspase-3 expression was observed in this study which indicates increased sperm apoptosis in diabetes. 28-days treatment with *C. borivilianum* root extract to diabetic rats resulted in higher percentage of HOS tail-coiled sperm and lower percentage of abnormal sperm as well as sperm with various abnormal shapes. The reduced in percentage of HOS-tail coiled sperm which indicate inability of the sperm to display tail-coiled appearance upon placing into hypoosmotic solution [44] suggest reduced integrity of flagella membrane [56]. Besides membrane integrity assessment, HOS tail coiled test is also useful to evaluate sperm viability [57]. The reduced sperm viability which was parallel to the reduced appearance of HOS-tail coiled sperm has been reported in both diabetic rodents [19] and humans [5]. Our finding which indicates reduced expression of caspase-3 in sperm of diabetic rats following *C. borivilianum* root extract treatment suggested that increased sperm viability was due to decreased in apoptosis. We speculated that the reduced appearance of HOS tail coiled sperm in diabetic rats could be due to increased in caspase-3, however this need further confirmation.

Diabetes induces oxidative-stress has been reported to cause peroxidation of sperm membrane lipid which might interfere with membrane fluidity and transport processes [58]. In view of this, appearance of various abnormal sperm shapes could be due to abnormal membrane or cellular and nuclear changes induced by diabetes [59]. More studies are needed to elucidate mechanisms underlying abnormal sperm appearances in diabetes. Treatment with *C. borivilianum* root extract prevents the increase in the amount of sperm lipid peroxidation as well as the levels of superoxide and nitric oxide free radical in sperm of diabetic rats. In both diabetic rats [19] and humans [60], lipid peroxidation was the major cause for sperm damage. Administration of *C. borivilianum* root extract to diabetic rats alleviates oxidative stress via several mechanisms which include reduced amount of free radicals such as superoxide and nitric oxide and preservation of total antioxidant capacity via maintaining near normal activity level of endogenous antioxidant enzymes. Additionally, a strong *in-vitro* free radical scavenging activity of the root extract further contribute towards the *in-vivo* free radical scavenging effects. The later effects may be attributed to higher amount of total phenolic content in the root extract as revealed by FTIR spectroscopic analysis. Meanwhile, ability of *C. borivilianum* root extract to lower FBG and HbA1c levels in diabetic rats could also help to reduce the risk of acquiring abnormal sperm morphology and characteristics and sperm oxidative stress [55, 59]. Scarano et al. [61] reported that sperm counts in diabetic rats was diminished following short-term exposure to hyperglycemia, while Amaral et al. [62] reported that prolonged hyperglycemia in rats adversely affect sperm concentration and motility due to oxidative stress.

## Conclusion

The root extract of *C. borivilianum* is potentially useful for male infertility treatment via overcoming impairment in sperm characteristics and via reducing percentage and appearance of abnormal sperm in diabetes. Additionally, the root extract also prevents elevation of sperm oxidative stress that might trigger apoptosis. Our findings therefore justify the claimed beneficial effects of *C. borivilianum* root extract on sperm in diabetes.
